# The Mobile Hospital delivers outreach acute care to nursing homes: healthcare staff interviews

**DOI:** 10.1186/s12245-026-01392-x

**Published:** 2026-09-25

**Authors:** Perttu Kontunen, Paulus Torkki, Maaret Castrén, Anu Helkkula

**Affiliations:** 1 Department of Public Health, University of Helsinki, Haartmaninkatu 8, PL 63, Helsinki, 00014 Finland; 2 Department of Emergency Medicine and Services, Helsinki University and Helsinki University Hospital, Helsinki, Finland; 3 Department of Industrial Engineering and Management, Aalto University, Espoo, Finland; 4 The Centre for Relationship Marketing and Service Management, Hanken School of Economics, Arkadiankatu 22, Helsinki, 00100 Finland

**Keywords:** Qualitative, Interviews, Nursing home, Outreach, Acute care, Staff

## Abstract

**Background:**

Nursing home residents often face unnecessary risks when visiting the emergency department (ED). Due to the limited rapid response capabilities of primary care and lack of equipment for acute illnesses, onsite specialized outreach units present a novel alternative. The Mobile Hospital is a nurse-led outreach unit providing onsite acute care to nursing home residents with remote physician support. The unit operates in the suburban area of Espoo, Finland. This study aimed to evaluate the Mobile Hospital from the perspective of healthcare staff.

**Methods:**

We interviewed nursing home nurses, emergency medical services (EMS) nurses, emergency physicians, and Mobile Hospital nurses, totaling 21 interviews. We employed inductive thematic analysis to analyze the interviews.

**Results:**

The Mobile Hospital was seen as a holistic way to deliver basic hospital-grade treatment to nursing homes, thus avoiding the risks involved in ED visits. However, it also introduced new dependencies on clinical expertise, teleconsultation support, and system coordination, underlining both its potential and its operational challenges. Successful adoption requires careful implementation and ongoing education of stakeholders regarding acute care protocols. Complementing the qualitative findings, the staff Net Promoter Score (NPS) for the Mobile Hospital was 81 (17 promoters, four passives, no detractors).

**Conclusion:**

The Mobile Hospital can be described as an interprofessional missing link in the acute care of nursing home residents, which the healthcare staff perceived as a promising model for avoiding unnecessary ED visits when implemented with adequate expertise and system support.

**Clinical trial number:**

Not applicable.

**Supplementary Information:**

The online version contains supplementary material available at https://doi.org/10.1186/s12245-026-01392-x.

## Background

Nursing home residents often have chronic and advancing illnesses, such as moderate to severe dementia, which cannot be curatively treated [1]. Frailty associated with these chronic illnesses impairs the capacity to recover from acute illnesses, injuries, and straining treatments [2]. When faced with an acute illness, these residents almost always require transportation to the emergency department (ED), a process that can be both straining and inefficient compared to outreach care. Advanced planning of treatments that benefit the individual is recommended [3]. These advance care plans, made together with residents living in nursing homes and/or their relatives, appear to enhance care quality [4] and facilitate onsite treatment for acute complaints that are not life threatening [5, 6].

Residents living in nursing homes need treatment at three levels of urgency: life threatening, acute, and non-urgent. Emergency medical services (EMS) are intended to deliver life-threatening care, while non-urgent care (and some acute care) is provided by nursing home staff, general practitioners (GPs), and outpatient clinics. EMS is not the optimal service for treating acute illness, injury, or exacerbation of a disease process that does not require resuscitation or immediate hospital transfer. Acute problems are relatively rare in nursing homes, and nurses may not maintain a sufficient level of expertise in these situations [1]. Additionally, GPs may be unavailable and lack the necessary equipment to treat acute problems, leaving EMS and ED visits as the primary options for addressing the most acute issues. Several factors contribute to ED transfers from nursing homes, including limited staffing capacity, fear of working outside a nurse’s capacity, lack of collegial support, communication problems between healthcare staff, and variable perceptions of advance care planning documents [7–9].

These challenges could be mitigated by implementing acute outreach units providing acute care specialized for nursing homes. Such units have the potential to save resources and reduce costs [10–12]. A recent systematic review indicated that acute outreach units might decrease ED visits and hospitalizations from nursing homes in (universally funded) healthcare systems, particularly when preventive components such as advance care planning and consultation services are included [13]. The demanding nature of acute care communication and decision-making in nursing homes, coupled with the stress nurses face in prioritizing care tasks, especially with limited knowledge and language skills, further underscores the need for such units [14, 15]. A 2022 interview study found that registered nurses in nursing homes noted that emergencies and ED visits could compromise the monitoring of other patients due to limited nursing staff on shift [16]. Providing collegial and medical support might empower and improve nursing home staff in acute care [17]. According to healthcare staff, examples of conditions that can be treated in nursing homes by outreach units are catheter problems, dehydration, falls, and infections [18, 19].

The Mobile Hospital is an acute outreach unit that operates in the city of Espoo in southern Finland which has approximately 290,000 inhabitants. The city of Espoo founded the Mobile Hospital in 2019 in close cooperation with the Helsinki University Hospital (HUS) to provide acute care to people living in nursing homes (and later to those in long-term home care). A significant reduction was seen in non-urgent EMS missions to nursing homes [20], ED visits, primary hospital episodes and estimated costs after the initiation of the Mobile Hospital [21].

In the city of Espoo, the transition to publicly subsidized nursing homes is based on the necessity for continuous assistance in activities of daily living (ADL) and nursing homes maintain their dedicated physicians who conduct monthly visits and remain accessible by phone during regular office hours. Additionally, an on-call physician is available around the clock for nursing homes. Typically, one to three registered nurses collaborate with practical nurses and nursing assistants within these facilities. It is mandatory for a nursing home physician to initiate the formulation of care plans or advance care plans shortly after a resident’s admission to the nursing home.

The catchment area of the Mobile Hospital in the city of Espoo includes 38 public, for-profit, and non-profit nursing homes. The mean number of residents per nursing home is 35, with a total of 1,342 residents. Almost all these residents receive publicly subsidized nursing home care—the most common form of nursing home residency in Finland. The Mobile Hospital works under the home hospital unit, which has traditionally provided healthcare after an ED visit or hospital stay, administering intravenous antibiotics and providing palliative care. The Mobile Hospital has partially taken the role of the EMS and the ED to simplify the care pathway. The aim is to help residents receive care in their own nursing homes and to reduce crowding in EDs. The Mobile Hospital has one specially trained registered nurse on shift 24/7, taking calls from nursing homes, giving advice, and going onsite when needed. The Mobile Hospital vehicle is built in a sport utility vehicle (SUV) with a custom metallic chassis for medicine boxes, fluids, and point-of-care (POC) test equipment. The most common medicines used include oral antibiotics (e.g., penicillin and trimethoprim) for urinary or respiratory infections, intravenous antibiotics (most commonly cefuroxime), and pain and palliative medication (morphine and oxycodone). The most common POC tests are C-reactive protein (CRP) and urinary samples. The Mobile Hospital nurse can consult the nursing home physician or the home hospital physician during office hours and the ED physician at other times.

Healthcare staff in nursing homes, EMS, and EDs form the frontline of acute care for people living in nursing homes and encounter most of the acute problems that the Mobile Hospital is planned to solve. This staff treats residents living in nursing homes and sees the effects of the Mobile Hospital on their organization and is therefore crucial to interview to gain insight into how the Mobile Hospital works.

There remains limited evidence on the effectiveness, cost-effectiveness, and staff perceptions of outreach units designed to deliver ED-grade acute care to nursing homes. Existing literature focuses mostly on end-of-life care planning or reducing hospital admissions through preventive measures [12] rather than providing immediate, hospital-quality acute care in the nursing home setting. There also remains limited qualitative evidence on how acute outreach service models reshape interprofessional collaboration and care pathways in practice. In particular, little is known about how these models are experienced across organizational boundaries (by different healthcare stakeholders), including EMS, ED and nursing homes.

This study addressed this gap by exploring healthcare staff’s experiences of a new outreach model—the Mobile Hospital—thereby providing insights into how healthcare staff perceive its effects on care coordination, ED transfers, and staff support.

Specifically, we focused on:

1. Investigating how various healthcare staff experience the Mobile Hospital’s integration into the current care system.
2. Exploring if implementation of the Mobile Hospital has led to a change in the quality of acute care as experienced by the staff involved.
3. Identifying benefits and challenges of the Mobile Hospital.

In this study, we followed the Institute of Medicine definition of care quality [22]. The results of this study were intended to provide insights into the Mobile Hospital model on acute care delivery.

## Methods

### Design

We conducted semi-structured in-depth interviews with healthcare staff working in different roles and organizations in the city of Espoo, nursing homes, and HUS who were all involved with the Mobile Hospital in treating nursing home residents. The interviews took place in 2020. We used the Consolidated Criteria for Reporting Qualitative Research (COREQ) checklist [23] to ensure transparent and comprehensive reporting. The interview guideline was iteratively created by the senior researchers in close collaboration with the junior researchers, informed by thorough review of the literature, and ensuring the questions were open-ended and designed to elicit neutral in-depth responses. The interview questions are listed in Table 1. We intentionally excluded direct questions about strengths and challenges to allow themes to emerge naturally from the participants’ perspectives without the influence of predefined categories. The first EMS interview was a pilot interview, and no changes were made to the questionnaire. Therefore, the pilot interview was used in the final study. Owing to the COVID-19 pandemic, this research focused on staff and was conducted by phone. Repeated contacts or comments on the interviews were not made (excluding the pilot participant, who wanted to elaborate on the benefits of the Mobile Hospital with one sentence by email afterward).

**Table 1:** Questions for semi-structured interviews

1.	How do you experience the service provided by the Mobile Hospital? What additional value, if any, does the Mobile Hospital generate?
2.	What value does a patient receive from a visit by the Mobile Hospital compared to a visit from Emergency Medical Services?
3.	Does the patient receive the necessary help for their acute problem from the Mobile Hospital, or is there a need for follow-up by Emergency Medical Services?
4.	What are your wishes or expectations regarding the services of Emergency Medical Services or the Mobile Hospital?
5.	What other important information can you tell about the Mobile Hospital?
6.	On a scale from 0 to 10, how likely are you to recommend the Mobile Hospital to residents with acute problem in nursing homes? (Net Promoter Score)

### Sampling and recruitment

We chose participants for our study by asking managers to send out our email invitations to the staff in their units. We made it clear that joining the study was voluntary, with no rewards or penalties involved. Physicians from ED; registered nurses from EMS, Mobile Hospital, and nursing homes; and practical nurses from nursing homes were included. The participants heard the questions for the first time during the interview. All participants signed an informed consent form. No relevant prior relationships with the researchers were present. None of the personally contacted participants declined. Most respondents were interviewed at their workplace (there is no information about whether other people were present). The interviews were conducted in the Finnish language. A demographic questionnaire was completed during the interviews (Table 2).

### Reflexivity

Interviews were conducted by two people: researcher P.K., a physician specializing in geriatrics, and a research nurse (P.Kos., a registered nurse, see Acknowledgements) with comprehensive, long-term experience in quantitative research data collection. Both were trained by senior researcher A.H., who is experienced in qualitative research and conducting semi-structured interviews. The training consisted of presentations, discussions, and training interviews. Senior researcher M.C., who is experienced in qualitative research, conducted the pilot interview together with P.K. to ensure the fidelity of the interview process. Throughout the study, discussions with the whole study group were held to enhance the depth of the data collected.

Three of the authors had prior involvement in evaluating the Mobile Hospital: P.K., P.T. and M.C. were involved in quantitative evaluations of the same Mobile Hospital. M.C. was working as head of department at HUS and therefore had an organizational interest in the development of an optimal acute care pathway. Before the interviews, the authors expected possible criticism from EMS staff owing to overlapping roles and different professional backgrounds. To manage these preconceptions, the interview guide avoided direct questions about strengths and challenges, and the interviewers P.K. and P.Kos. and supervising analyst A.H. were not involved in the development or operation of the service. A.H. reviewed the coding at both code and theme level, with particular attention to ensuring that critical and dissenting comments were retained in the analysis.

### Data analysis

All the interviews were transcribed verbatim by a professional agency. We used the qualitative data analysis software ATLAS.ti 9 for Mac (ATLAS.ti Scientific Software, Berlin, Germany) for coding. Researcher P.K. performed the initial coding under close supervision and comprehensive code- and theme-level evaluation by A.H. The whole research team regularly discussed the coding to ensure consistency and reflexivity, and discrepancies in interpretation were resolved through consensus.

We employed inductive thematic analysis [24] to identify patterns and themes within the interview data. This allowed themes to emerge from the data through a process of reading, coding, and categorizing. The analysis began with open coding, noting initial ideas and potential themes. Then we moved to a more focused phase, collating codes into potential themes and reviewing these in relation to the entire data set. This iterative process continued until we could name distinct themes that best describe the participants’ experiences. Our intention was to remain close to the data, ensuring that our findings were grounded in the participants’ perspectives without a predetermined theoretical bias. To complement the thematic analysis, we calculated a Net Promoter Score (NPS) as a descriptive indicator of participants’ likelihood of recommending the Mobile Hospital model. Following the standard NPS convention, participants scoring 9–10 on the 0–10 scale were classified as promoters, 7–8 as passives, and 0–6 as detractors. The NPS was calculated as the percentage of promoters minus the percentage of detractors. Because the NPS was obtained from a small purposive sample, it is reported only as a descriptive addition to the qualitative findings.

### Ethical considerations

Sensitive or personal information was not sought during the interviews. Participation was voluntary, and the managers had no extraneous incentives other than to assist in the study of the Mobile Hospital. The participants signed an informed consent form and were allowed to withdraw from the study without any consequences. This study received ethical approval from the Ethics Committee of Helsinki University Hospital (HUS/2790/2019).

After writing the initial manuscript version, we used the Artificial Intelligence tool Paperpal (2002–2023 Cactus Communications) and ChatGPT 4 (2024 OpenAI) to make the text more fluent.

### Participants

The data included 21 interviews with seven nursing home nurses (one of them in a managerial position overseeing the nursing home), six EMS nurses (one of them being the department chief), three emergency physicians (one of them being the head of the ED department), and five Mobile Hospital nurses. Two interviews were paused because of the urgent care situation and were continued later. The duration of the interviews ranged from 7 to 54 min (mean 14.6 min, median 12.0 min, standard deviation 9.9); this duration did not include study briefing and introduction. Short interviews occurred in all stakeholder groups and included concise but complete answers to all questions. These interviews contributed to the same themes as the longer interviews. Each subsequent question was asked only after the participant no longer brought up new content in response to follow-up questions. Theme saturation was monitored across the complete dataset (not within stakeholder groups) during data acquisition. After every one to three interviews, P.K. and A.H. reviewed the preliminary codes for new codes or themes, and saturation was judged to have been reached after 13 interviews, when no new themes emerged. A total of 21 interviews were nevertheless conducted, consistent with a pre-specified threshold of at least 20 interviews, and to ensure that all four stakeholder groups were represented. The pilot interview (with an EMS nurse) was conducted at a hospital in person by P.K. and M.C. Of the remaining 20 phone interviews, 16 were conducted by P.Kos. and four by P.K. Table 2 presents the participants’ characteristics.

**Table 2:** Interview participant characteristics

Characteristic	Nursing home nurse *N* = 7	EMS nurse *N* = 6	Emergency physician *N* = 3	Mobile Hospital nurse *N* = 5
Mean age (SD)	46 years (6.2)	37 years (3.4)	37 years (*)	44 years (12.8)
Gender (F: M)	6:1	0:6	0:3	4:1
Working experience in mean years (SD)	18 years (9.6)	12 years (5.2)	6 years (*)	16 years (13.5)
Job description (Only fieldwork: Partial administrative work)	6:1	5:1	2:1	5:0

Standard deviation = SD

* SD not shown for emergency physicians due to small number of participants

## Results

In the interviews, we identified four primary themes that portray the participants’ experiences regarding the Mobile Hospital: change of care practices, communication, relations to other healthcare, and benefits and challenges. Supplementary Fig. 1 presents the elaborated coding tree to illustrate the systematic approach we employed in the analysis. This visual representation clarifies the hierarchical structure of the thematic categories.

### Change of care practices

According to the participants, the Mobile Hospital is equipped and able to handle most acute care situations in nursing homes. This approach changes the usual care practices, as nursing home staff now have dedicated access to acute care support. Nursing home nurses appreciated that the Mobile Hospital is especially aware of older adults’ acute complaints and has the readiness to treat most of them, with applicable training and focus.

Emergency physician (29 years old) highlighted the effectiveness of the Mobile Hospital’s support: “I have experienced that the nurses who consult [from the Mobile Hospital] are very experienced and have good equipment, so a situational picture can be formed surprisingly well on the phone: what is going on in the nursing home [acute care situation]. And I can trust that they know what they are doing and that the information they provide is accurate.”

Mobile Hospital nurse (63 years old) emphasized the comprehensive approach they take: “Then we consider the whole picture more broadly…How she [the resident] is doing in the nursing home, how she understands the situation, and how the [nursing home] nurses understand the situation. We see the whole picture better than the ED does during a resident’s visit.”

The NPS for the Mobile Hospital was 81 (17 promoters, four passives, no detractors). The NPS describes how likely the participants were to recommend the Mobile Hospital to residents living in nursing homes. Two EMS nurses, one nursing home nurse, and one Mobile Hospital nurse were passives, meaning they gave a score of eight.

### Communication

In addition to caring for the resident, the Mobile Hospital educates nursing home staff to recognize illnesses and perform initial evaluations to provide concise and relevant information during consultations. Most nursing home nurses felt relieved to receive warm collegial help.

The Mobile Hospital has well-established connections to the home hospital and has the know-how for what to do and who to call when an acute illness is not treatable by the Mobile Hospital. Interprofessional communication and trust are important for acute care of residents living in nursing homes. Siloed healthcare providers were seen as a problem by some participants. Respectful and helpful encounters with nursing home staff by EMS or the Mobile Hospital were an important topic.

Nursing home nurse (45 years old) described the challenges faced with the EMS: “Sometimes, it is like a battle with the EMS; they do not take us seriously (that there is something wrong with the patient).”

Participants felt that the Mobile Hospital overcame some of these barriers by working in a niche that has not been filled before.

### Relations to other healthcare

Based on the interviews, nursing home staff appeared to be the healthcare staff group that benefits most from the Mobile Hospital. Participants described that having someone to lean on in an acute care situation relieves the stress of acute care decisions and follow-up, especially during night shifts. Some nursing home nurses have imperfect language skills and may work alone or almost alone in a shift. The Mobile Hospital was considered particularly useful in these situations.

Nursing home nurse (45 years old) said: “The Mobile Hospital brings a lot of added value. I receive good collegial support from a Mobile Hospital registered nurse when I am alone on a shift.”

Participants felt that the care received is no longer solely in the hands of a nursing home nurse and that the Mobile Hospital therefore makes care more equitable between nursing homes. Two nursing home nurses and the Mobile Hospital nurses indicated that after the Mobile Hospital consultations and visits, the nursing homes started to develop their own consultation skills and preliminary diagnostic capacity.

Nursing home nurse (37 years old) mentioned: “Especially inexperienced [nursing home] nurses need support and safety so that they always have a source to ask for advice.”

According to the participants, this educational aspect may improve the ability of the healthcare system to respond to acute complaints. Nursing homes may be better able to take care of basic acute complaints, hasten the right care, and consult with the Mobile Hospital, EMS, and ED.

Nursing homes have a culture of social care—housing, ADL, and caring relationships are their focus. EMS, on the other hand, focuses on clinical decision making and standardized care pathways, depending on the specific illness or injury. The Mobile Hospital is a link between these two aspects. The Mobile Hospital “speaks ED language” and helps deliver information about the patient status in the same terms and background information as the doctors and ED nurses.

One participant talked about the kindness of the Mobile Hospital staff. She was more eager to call the Mobile Hospital than the “cold and distant” EMS, allowing the resident to stay in the familiar nursing home with familiar staff rather than leaving them “to the outer world” with unfamiliar people.

Participants perceived that when the Mobile Hospital delivers care, other healthcare sectors can focus on their core competencies. EMS can focus on life-threatening situations, evaluation, stabilization of the patient, and fast transfer to the ED with shorter waiting times. Routine laboratory tests can be performed in laboratories and nursing homes. The ED can focus on demanding evaluation and the initiation of specialized treatments, and the hospital ward can provide treatments that cannot be delivered in a nursing home. In the participants’ view, the Mobile Hospital fills a gap in the acute healthcare system without adding an unnecessary layer of care. Consistent with these perceptions, the interviewed healthcare staff reported a favorable NPS.

### Challenges and limitations of the model

While the Mobile Hospital was widely perceived as a valuable addition to the acute care system, participants also described challenges. These included uncertainty in triage accuracy and clinical decision-making in onsite settings, reliance on remote physician consultation, and the need for advanced competencies among Mobile Hospital nurses. The participants noted that the model requires skilled staff and good coordination across organizational boundaries.

Two EMS nurses thought there was a risk that nursing homes might neglect acute care competence because they could outsource acute care to the Mobile Hospital. However, no such examples were reported.

EMS nurse (39 years old) stated: “Outreach units should not substitute for basic care competence that the nursing home should possess.”

One emergency physician and one EMS nurse expressed worries that consultation-based care or telemedicine would be taken too far, where acute situations are not diagnosed correctly, causing patients to suffer harm. Care plans should be in place to enable timely treatment; otherwise, the patient may receive unnecessary burdensome diagnostic testing and treatment. This is in the hands of primary care physicians and nursing homes and should be demanded by authorities. Ongoing education about the Mobile Hospital is needed because of changing staff. Reinforcing (repeating) information helps all stakeholders to contact the Mobile Hospital smoothly in acute situations.

EMS nurse (35 years old) suggested: “Some kind of briefing or presentation about the Mobile Hospital would be welcome, like a video that explains what it does.”

The Mobile Hospital nurses sometimes had problems contacting consulting physicians. Therefore, a physician dedicated to the Mobile Hospital was requested. The evaluation of a new patient with an acute complaint is a demanding task, and the Mobile Hospital nurses need sufficient education and, preferably, experience. The Mobile Hospital nurses work alone, so they do not usually receive instant feedback, and double-checking is not possible.

Another challenge is that the Mobile Hospital is not designed to address social issues, such as loneliness or anxiety caused by social isolation. The participants commented that a single Mobile Hospital visit cannot fully tackle such ongoing psychosocial concerns. The participants recognized that psychosocial concerns are also a challenge for the regular EMS-ED-based acute care system. Therefore, access to primary care is crucial.

**Fig. 1:**
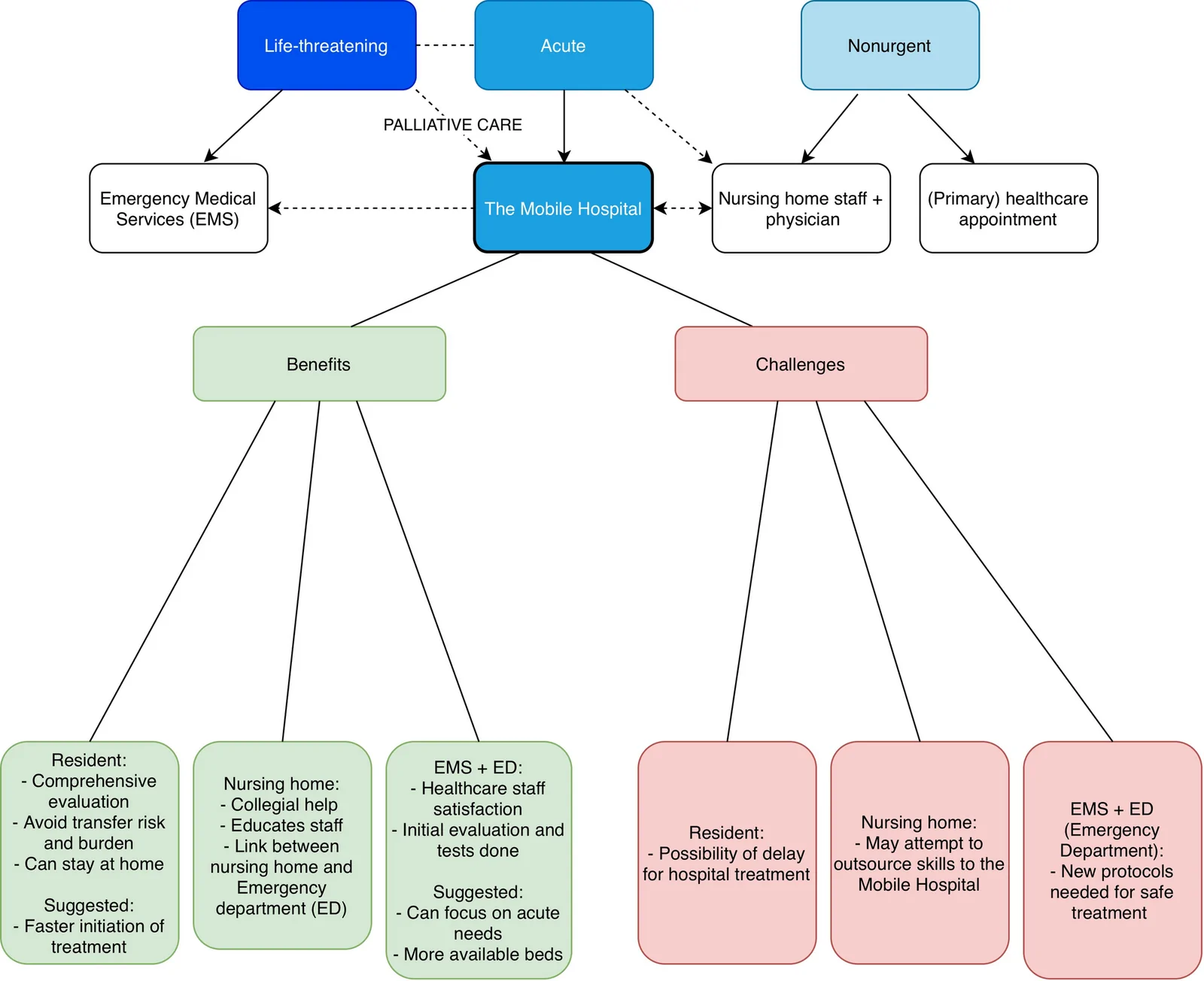
Urgency of care in nursing homes—Mobile Hospital benefits and challenges (conceptual synthesis)

The participants noted that most injuries resulting in a decrease in functional capacity required an ED visit. Two participants saw insufficient language skills as a problem in nursing homes, but this was not related to the Mobile Hospital, although it might affect the applicability of the outreach care model. Figure 1 summarizes the benefits and challenges that the participants identified with the Mobile Hospital. The figure is a conceptual synthesis of the perceived benefits and challenges derived from the interviews, not a formal clinical pathway.

## Discussion

The challenge in nursing home care research to date has been how to provide optimal value-based acute care to residents, considering the benefits and harms of treatment. The Mobile Hospital was implemented to bridge this critical gap: ensuring timely, appropriate acute care for nursing home residents without the strain and risks associated with ED visits.

This qualitative study complements earlier quantitative evaluations of the Mobile Hospital model that demonstrated reduced (non-urgent) EMS missions, ED visits, primary care hospital episodes and acute care costs [20, 21]. This interview study did not measure these outcomes and cannot independently demonstrate changes in ED use, costs or safety. Rather, it describes how healthcare staff perceive the Mobile Hospital outreach model to work in practice, which professional and system-level mechanisms they consider important, and what changes they attribute to it.

To the nursing home acute care and geriatric nursing literature, this study adds evidence that healthcare staff consider the Mobile Hospital to be a good improvement. In the staff’s experience, the Mobile Hospital streamlines the acute care pathway while still providing basic hospital-grade diagnostics and treatment. The staff experienced that the residents received more holistic care from the Mobile Hospital than from the regular acute care pathway (EMS and ED). Taken together, the interviews portray the Mobile Hospital as the missing link in acute care for nursing home residents.

Another important finding is that ongoing education about the acute care pathway is seen as crucial for the Mobile Hospital and the entire acute care process to function effectively. Emails, easily readable leaflets, available phone numbers, and understandable instructions about when to call the Mobile Hospital are useful for nursing home nurses in acute care situations. Extrapolating from a recent study on nursing home care quality improvement, the implementation of the Mobile Hospital and other outreach acute care pathways might be more successful if the nursing homes employ at least one nurse with a higher level of education [25].

These findings are useful to other acute outreach nursing home units that resemble the Mobile Hospital, especially in capitation-funded healthcare systems, with suburban or urban distances between nursing homes (several dozen kilometers between nursing homes might affect the results).

It is a general perception that trust and coordination among stakeholders (EMS, ED, the Mobile Hospital, and nursing home staff) are necessary. Effective communication and appreciation of professional roles are pivotal for patient-centered collaborative care [26]. In this case, trust in nursing home staff is especially pivotal. According to the participants, the Mobile Hospital can educate nursing home staff and work as a “communication link” between the nursing home staff and EMS/ED and therefore overcome some of the barriers to efficient interprofessional care. This finding aligns with results from a Swiss study, where nursing home nurses reported feeling accompanied and relieved of their care burden due to inclusive support from an interprofessional mental health outreach service [27]. In line with studies by Bandurchin and Codde [18, 28], the outreach unit (similar to the Mobile Hospital) was seen as an outstanding communication link between the nursing home and the ED. Nursing home nurses and nursing assistants seem to be willing to take more responsibility for the acute evaluation of residents, according to an Australian interview study [29]. A recent Danish focus-group interview study on outreach services to nursing homes concluded that such a model enhanced the dignity of care provided to residents, particularly by tailoring acute care to their wishes and capabilities [30].

An important aspect of the Mobile Hospital is the generally favorable experience of interviewed participants, who noted that the nursing home nurses receive collegial support. It is possible that this easily accessible and dedicated service manned by nurses working “between the healthcare silos” is able to overcome unhealthy communication styles. We propose that this collegial and education-providing service model could counter repressive and even dangerous ways of interaction between nurses and doctors/consultant nurses [31] and promote work enthusiasm of nursing home nurses [32]. It is evident that competent support from nurse colleagues promotes nurses’ job satisfaction [33].

According to our interviews, the most important characteristics of the Mobile Hospital seemed to be competence in acute care and the ability to make informed decisions and communicate with the nursing home, ED, and primary care for next steps. In addition, the participants provided positive comments about the Mobile Hospital’s equipment, POC tests, and procedures.

The Mobile Hospital is not suitable for life-threatening situations. Primary care is also needed to continue acute treatment and provide non-urgent care and care planning. Instructions on when to call the Mobile Hospital must be available in nursing homes. As noted earlier, education about why, when, and how to contact an acute outreach unit, such as the Mobile Hospital, needs to be repeated frequently. A recent review of Community Paramedicine services (partially acute outreach care) proposed that it is important to carefully design and locally implement a new interprofessional service to avoid duplicate work and resistance from established provider groups [34].

Acute outreach unit care services are relatively new. Residents living in nursing homes deserve care that focuses on quality of life. *Primum non nocere (in Latin)*—“first, do no harm”—is a good starting point for providing care to residents living in nursing homes. It is harmful to transfer a frail sick person to a strange place with a strange person if the resident can receive better or sufficient care in the nursing home. Transferring to the ED often does not produce value for the resident if an outreach service that functions comparably to the Mobile Hospital is available.

Participants perceived the Mobile Hospital as complementing EMS, hospital, and primary care to provide holistic care. The model’s cost-effectiveness was not assessed in this study but has been examined in an earlier quantitative evaluation [21]. The 24/7 availability of the Mobile Hospital may account for many of the care access benefits. Nursing home nurses reported that its availability reduces the burden of being alone on a shift, possibly with a low level of language skills. Participants also perceived that the Mobile Hospital allows EMS and the ED to focus on their core competencies and care in life-threatening situations.

We also found that the Mobile Hospital nurses shared similar views about the role of the Mobile Hospital with other healthcare staff. New possible benefits were identified in this study: the Mobile Hospital can do more than treat an imminent acute illness; and the Mobile Hospital can advise nursing homes on how to continue care after the initial encounter. In our study, a possible concern about nursing homes neglecting their own acute care competency was raised as a new theme.

### Strengths and limitations of the work

First, this study was conducted in a single urban Finnish setting, which may limit transferability.

Second, as with all qualitative research, the findings are interpretive and context dependent. Third, recruitment may have introduced selection bias. Managers distributed the invitations to participate, which allowed managerial gatekeeping, and participation was voluntary. The strongly favorable NPS and the generally positive attitudes could partly reflect self-selection of staff with more positive experiences of the Mobile Hospital. We sought to mitigate this by including four stakeholder groups, asking questions without predefined strengths and challenges, and by actively coding critical comments, but the possibility of volunteer bias remains and the NPS should not be generalized to the wider healthcare staff.

Before conducting the interviews, we expected some criticism from the EMS staff since the Mobile Hospital may overlap with their service. However, no such effect was observed. We informed the participants that their interview responses would be confidential, and we do not believe that the environment that the participants were in (such as a nursing home or ED) while taking part in the interview had a significant impact on the results. We used semi-structured interviews, which are flexible and allow for open-ended responses. This approach gave participants the opportunity to discuss topics that were not strictly about the Mobile Hospital but were still relevant to the broader context of acute care in nursing homes. By doing so, we were able to gain a more comprehensive understanding of how acute care is provided in nursing homes, considering various factors and perspectives beyond just the Mobile Hospital.

Theme saturation was judged to have been achieved across the complete dataset, but not necessarily within each stakeholder group. We do not think that pausing an interview due to an urgent care situation influenced the outcome. One pause occurred at the interview’s outset, and the other took place after the participant had already expressed their initial view about the Mobile Hospital and was providing additional elaboration. The participants did not receive any revenue from the Mobile Hospital (excluding the Mobile Hospital nurses’ normal monthly salary), nor did we identify any other significant incentives to appraise the Mobile Hospital positively. The EMS staff, nursing home nurses, emergency physicians, and Mobile Hospital nurses had very similar attitudes toward the Mobile Hospital. Therefore, the interviews with the different groups supported each other. We conducted this study in a single Nordic country with mostly publicly capitation-funded nursing home care and municipality-funded acute care and hospital services, which may limit the transferability of the findings, particularly to countries with very different acute care or healthcare funding systems. Nursing home physicians visited nursing homes in the city of Espoo usually only once a month, which may give room to the Mobile Hospital to treat more acute situations than in a model where a physician is available in the nursing home once a week or more often. However, physicians do not usually carry POC test equipment or intravenous medications like the Mobile Hospital does.

The transferability of the Mobile Hospital model depends on specific contextual features: 24/7 physician consultation availability, EMS-ED services integration, and the staffing pattern of nursing home services (in Finland registered nurses working with practical nurses, often alone on night shifts), and the availability of point-of-care testing and stocked medications. Systems with fragmented care coordination or limited prehospital resources may face greater implementation challenges. Our findings offer insights into integrated acute care, but their application requires careful consideration of local system conditions.

In this study, we did not interview the residents living in nursing homes. The interview recruitment process and interviews took place during the COVID-19 pandemic in 2020, and the nursing homes prioritized regular care over organizing resident interviews; therefore, in-person interviews were not possible. In addition, most residents living in nursing homes have moderate to severe dementia [1], which challenges the reliability of their interviews. If an acute outreach unit to nursing homes is initiated, we recommend interviewing nursing home residents in person immediately after the outreach care visit.

## Conclusion

The focus of the Mobile Hospital is to increase the value of care for residents living in nursing homes. This study shows that the interviewed healthcare staff believe the Mobile Hospital to fulfill this goal. In the staff’s view, the Mobile Hospital allows residents to stay in their nursing homes, improves the quality of acute care, and offers collegial support to nursing home staff. Whether the Mobile Hospital reduces ED and hospital ward use was not examined in this study. Earlier quantitative evaluations of the same model have reported reductions in EMS missions, ED visits, primary care hospital episodes and acute care costs [20, 21].

Our study provides an in-depth qualitative evaluation of a new acute outreach service, contributing to the literature gap on how frontline healthcare staff perceive and interact with such service units. The Mobile Hospital can be described as an interprofessional missing link in acute care to nursing homes. Our study helps to understand and develop the role of acute outreach services in the healthcare system.

## Recommendations for further research

If an outreach unit service, such as the Mobile Hospital, is initiated in a different healthcare system, we recommend ongoing education of the acute care pathway to healthcare and social care stakeholders such as nursing home staff. The service would then, optimally, be examined scientifically using a mixed-methods study setting to assess the effectiveness and safety of the service, preferably with a cluster-randomized or quasi-experimental research design with cost analysis and interviews.

## Supplementary Information

Below is the link to the electronic supplementary material.

Supplementary Material 1: supplementary figure 1: Themes and subthemes—the clarified coding tree

## Data Availability

The study data are available if permission is requested and granted from Helsinki University Hospital (HUS) and the Western Uusimaa Wellbeing Services County, and upon request from the corresponding author.

## Abbreviations

ADL: Activities of daily living
COREQ: Consolidated criteria for reporting qualitative research
CRP: C-Reactive protein
ED: Emergency department
EMS: Emergency medical services
GP: General practitioner
HUS: Helsinki university hospital
NPS: Net promoter score
POC: Point-of-care
SD: Standard deviation
SUV: Sport utility vehicle
